# Biogenesis and Lipase-Mediated Mobilization of Lipid Droplets in Plants

**DOI:** 10.3390/plants11091243

**Published:** 2022-05-05

**Authors:** Yun Ju Choi, Kseniia Zaikova, Soo-Jin Yeom, Yeong-Su Kim, Dong Wook Lee

**Affiliations:** 1 Department of Integrative Food, Bioscience and Biotechnology, Chonnam National University, Gwangju 61186, Korea; yoonjoo1523@naver.com (Y.J.C.); kseniiazaikova@yahoo.com (K.Z.); 2 School of Biological Sciences and Technology, Chonnam National University, Gwangju 61186, Korea; soojin258@jnu.ac.kr; 3 Wild Plants Industrialization Research Division, Baekdudaegan National Arboretum, Bonghwa 36209, Korea; 4 Department of Bioenergy Science and Technology, Chonnam National University, Gwangju 61186, Korea; 5 Bio-Energy Research Center, Chonnam National University, Gwangju 61186, Korea

**Keywords:** lipid droplet (LD), LD-associated proteins, oleosin, lipase, SDP1

## Abstract

Cytosolic lipid droplets (LDs) derived from the endoplasmic reticulum (ER) mainly contain neutral lipids, such as triacylglycerols (TAGs) and sterol esters, which are considered energy reserves. The metabolic pathways associated with LDs in eukaryotic species are involved in diverse cellular functions. TAG synthesis in plants is mediated by the sequential involvement of two subcellular organelles, i.e., plastids - plant-specific organelles, which serve as the site of lipid synthesis, and the ER. TAGs and sterol esters synthesized in the ER are sequestered to form LDs through the cooperative action of several proteins, such as SEIPINs, LD-associated proteins, LDAP-interacting proteins, and plant-specific proteins such as oleosins. The integrity and stability of LDs are highly dependent on oleosins, especially in the seeds, and oleosin degradation is critical for efficient mobilization of the TAGs of plant LDs. As the TAGs mobilize in LDs during germination and post-germinative growth, a plant-specific lipase—sugar-dependent 1 (SDP1)—plays a major role, through the inter-organellar communication between the ER and peroxisomes. In this review, we briefly recapitulate the different processes involved in the biogenesis and degradation of plant LDs, followed by a discussion of future perspectives in this field.

## 1. Introduction

Lipid droplets (LDs) are lipid-storage compartments universally present in most eukaryotes, as well as in archaea and prokaryotic bacteria [1]. The core of LDs, mainly composed of triacylglycerols (TAG) and sterol esters, is surrounded by a monolayer of amphipathic glycerolipids, which contain a variety of proteins required for the structural maintenance and metabolism of LDs [2,3]. Numerous studies have reported crucial roles of LDs in energy storage and as precursors for cellular membranes [1,4,5]. In addition, LDs are involved in different cellular physiological processes, including protein storage [6], pathogenesis of microorganisms [7,8,9,10], stress responses in plants [11,12], protection of the cells from endoplasmic reticulum (ER) stress [13,14], neutralization of lipotoxic fatty acids [15], reactive oxygen species (ROS) detoxification [16], and membrane trafficking [17,18].

Plants contain two types of LDs, i.e., plastoglobules, which are present in plant-specific organelles called plastids [19], and cytosolic LDs, which are derived from the ER [1,19,20]. Plastoglobules are present in all types of plastids. Among them, chloroplasts possess an intra-organellar membrane system called the thylakoid, to which the plastoglobules are connected [21]. Unlike cytosolic LDs, which are commonly present in most eukaryotes, plastoglobules are surrounded by a monolayer of galactolipids, which is the major polar lipid of the thylakoid membrane [22]. Various types of plastids, including etioplasts, chloroplasts, chromoplasts, and elaioplasts have been characterized, which can interconvert depending on various developmental and environmental conditions [23,24,25]. Plastoglobules are present in different types of plastids with varying size, morphology, proteome, and lipid contents, and collectively contribute to the specialized functions of each type of plastid [1,19], such as thylakoid biogenesis [21,26], isoprenoid metabolism [19], senescence [27], and jasmonate metabolism, among others [28,29]. Previous studies have discussed the involvement of enzymes associated with plastoglobules in the metabolism of isoprenoid lipids [19,21,30].

The characteristics of plant ER-derived cytosolic LDs are highly similar to those of other eukaryotic species, with respect to the composition and morphology of the cytosolic LDs, and their biogenesis mechanisms [1]. However, the plant cytosolic LDs have some unique characteristics, such as the intracellular location where lipid synthesis begins, plant-specific LD-associated protein factors, and the inter-organellar communication between LDs and the peroxisomes, which are the site of β-oxidation [31,32]. In this review, we aimed to recapitulate recent progress in the elucidation of the mechanisms involved in the biogenesis and mobilization of ER-derived cytosolic LDs in plants and provide future perspectives in this field.

## 2. Biogenesis of LDs in Plant Cells

**Figure 1:**
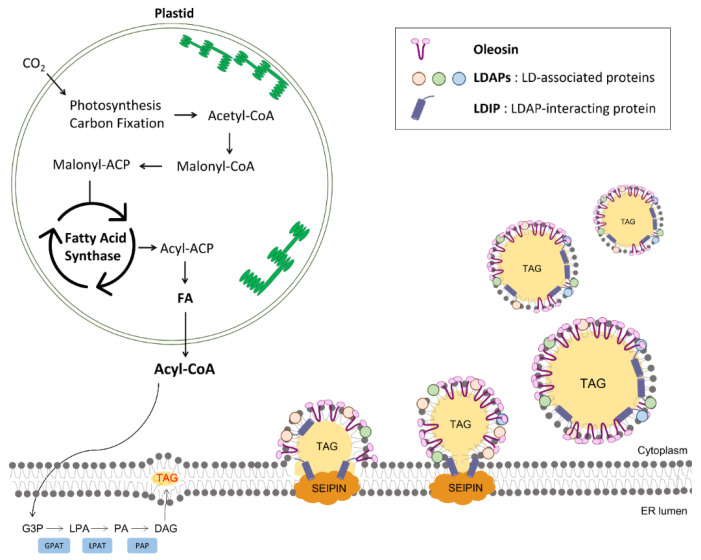
Biogenesis of lipid droplets in plants. ACP, acyl carrier protein; CoA, coenzyme A; G3P, glycerol-3 phosphate; GPAT, glycerol-3-phosphate-acyltransferase; LPA, lysophosphatidic acid; LPAT, lysophosphatidic acid acyltransferase; PA, phosphatidic acid; PAP, phosphatidic acid phosphatase; DAG, diacylglycerol.

Fatty acids (FAs) are the building blocks of membrane lipids and TAG. In animal cells, FAs are usually synthesized in the cytoplasm by fatty acid synthase (FAS), a multifunctional enzyme, which requires acetyl coenzyme A (acetyl-CoA) derived from the mitochondria [33]. However, most of the FAs present in plant cells are synthesized in the plastids of both photosynthetic and non-photosynthetic tissues (Figure 1) [22]. The acetyl-CoA synthesized in plastids is sequentially catalyzed to produce FAs by the action of two enzyme systems, acetyl-CoA carboxylase and FAS; FAS represents a prokaryotic enzyme system and, thus, forms a complex composed of four independent enzymes [34,35]. A proportion of FAs produced in the plastids is utilized for the synthesis of plastid galactolipids, such as monogalactosyldiacylglycerol (MGDG) and digalactosyldiacylglycerol (DGDG) [36]. The FAs destined for extraplastidic lipid assembly are first exported by the FATTY ACID EXPORT (FAX1) channel at the chloroplast inner envelope [37]. Detailed analyses of the *Arabidopsis* *fax1* loss-of-function mutant and FAX1-overexpressing lines revealed that FAX1 is crucial for the production of ER-derived TAG. In *fax1* knockout lines, the levels of several chloroplast-derived lipids increased, whereas those of ER-derived lipids decreased. Conversely, the FAX1-overexpressing plants accumulated higher amounts of ER-derived TAG than the wild-type plants [37]. Moreover, *Chlamydomonas reinhardtii* overexpressing two isoforms of *Chlamydomonas* FAX proteins harbored elevated levels of TAG [38]. The FAs exported through the FAX1 channel are conjugated to CoA by long-chain acyl-CoA synthetase (LACS9) at the outer envelope of the plastids to generate acyl-CoA, which is subsequently transported to the ER [39]. Previous studies have demonstrated that cytosolic acyl-CoA-binding protein 4 and 5 (ACBP4 and ACBP5) are involved in the transfer of acyl-CoA from the plastids to the ER [40,41].

In the ER, glycerol-3-phosphate is converted into TAG, via successive incorporation of acyl-CoA, by the Kennedy pathway (Figure 1) [42]. First, glycerol-3-phosphate undergoes acylation at the *sn*-1 position, which is mediated by glycerol-3-phosphate acyltransferase (GPAT). Next, the resulting lysophosphatidic acid (LPA) is linked to another acyl chain at the *sn*-2 position by lysophosphatidate acyltransferase (LPAT), thereby producing phosphatidic acid (PA). Following that, a proportion of PA is dephosphorylated by phosphatidic acid phosphatase to generate diacylglycerol, which is converted into TAG via acyl-CoA:diacylglycerol acyltransferases (DGATs). In addition to the Kennedy pathway, an alternative pathway, which synthesizes TAG through acyl-CoA-independent acylation of diacylglycerol, is involved. In this pathway, phosphatidylcholine functions as an acyl donor [42,43].

TAGs synthesized in the ER are then sequestered to form LDs at the ER-LD junctions primarily by the SEIPIN proteins (Figure 1) [44,45,46], which are universally present in all eukaryotes [44,45,47,48]. Previous studies on SEIPIN proteins from non-plant species have revealed that SEIPIN proteins coordinate the maturation of nascent LDs into mature LDs, thereby regulating the number and size of LDs [46,47,49]. Unlike humans and yeast, which possess only one SEIPIN protein, *Arabidopsis* harbors three homologs of the protein: SEIPIN1, SEIPIN2, and SEIPIN3 [45]. A previous study on yeast SEIPIN revealed that the N-terminal domain upstream of the first transmembrane domain of SEIPIN controls the size of LDs [48]. Among the three *Arabidopsis* SEIPIN proteins, SEIPIN1 harbors a notably shorter N-terminal domain than SEIPIN2 and 3. Intriguingly, deletion and domain swapping experiments using *Arabidopsis* SEIPIN1, 3, and yeast SEIPIN revealed the functional conservation of these N-terminal domains and their roles in decreasing the size of LDs [45]. As such, what are the role of the relatively long N-terminal domain of SEIPIN2 and 3? Recently, the membrane-tethering protein vesicle-associated membrane protein (VAMP)-associated protein 27-1 (VAP27-1) was identified to interact with the FFAT motif present in the N-terminal domain of SEIPIN2 and 3 [50]. *Arabidopsis* VAP27-1 is involved in tethering the ER membrane to the plasma membrane [51]. Interestingly, disruption of *VAP27-1* caused enlarged LDs, which was also observed in *seipin2*/*seipin3* double-knockout seeds, indicating that both VAP27-1 and SEIPIN 2/3 are required to mediate the proper biogenesis of LDs [50]. Unlike SEIPIN1, which is predominantly expressed in embryos, SEIPIN2 and 3 play critical roles not only in seeds, but also in pollen grains by contributing to efficient pollen germination and ovule fertilization [52]. In addition to SEIPIN proteins, plant-specific LD-associated proteins (LDAPs) were identified to play important roles in controlling the number of LDs (Figure 1). The overexpression of *Arabidopsis* LDAPs increased the number of LDs in leaves, whereas loss-of-function mutants with one or three disrupted *LDAP* genes displayed the opposite phenotype [53]. In addition, Pye et al. recently showed that another LD protein, LDAP-interacting protein (LDIP), interacts with SEIPIN as well as LDAPs to modulate the number and size of LDs in plants (Figure 1) [54,55].

Oleosin, one of the most studied LD proteins in plants, is abundantly present in the seed LDs and is crucial in preventing the coalescence of LDs during seed desiccation (Figure 1) [56,57]. Oleosins are co-translationally inserted into the ER membrane, where their N- and C-terminal regions are oriented toward the cytoplasm; these regions are thought to interact with the head groups of the phospholipid monolayer of LDs. Conversely, their internal hydrophobic domain is intercalated between two ER leaflets where TAGs are accumulated. This long hydrophobic hairpin arm containing a conserved proline-knot is crucial for the targeting of oleosins to ER-LDs and the extraction of budding LDs from the ER to the cytosol [58]. The loss-of-function mutants, with suppressed expression of *oleosin* genes, displayed enlarged LDs, altered lipid accumulation, and abnormal storage organelles. Consequentially, they exhibited the delayed germination phenotype, which might have resulted from the fusion of LDs with low oleosin levels [59]. These results indicate that oleosins are crucial for the proper biogenesis of LDs, as they stabilize and determine the size of seed LDs. In addition to oleosins, other LD-associated proteins, such as caleosin and steroleosin, have also been identified; they contain the hydrophobic hairpin that penetrates into the core of LDs filled with TAGs, which is similar to that in oleosins [60]. However, they have additional domains not present in oleosins; for example, caleosins act as peroxygenases, thereby catalyzing the hydroperoxide-dependent oxygenation of unsaturated FAs, which contributes to oxylipin metabolism, leading to various plant stress responses [61]. Steroleosin possesses a sterol-binding motif and can convert sterol substrates to brassinosteroids, which are important plant hormones required for controlling various stages of plant development [62,63]. Peramuna et al. successfully sequestered sesquiterpene patchoulol into LDs in *Physcomitrella patens* by overexpressing patchoulol synthase (PTS) fused to moss oleosin (*Pp*OLE1), *Arabidopsis* LDAP1, and moss SEIPIN (*Pp*Seipin325) [64]. Moreover, PTS fused to *Pp*Seipin325 resulted in increased LD size and patchoulol accumulation in cells [64].

## 3. Mobilization of LDs in Plants: Pivotal Roles of LD-Associated Lipases

**Figure 2:**
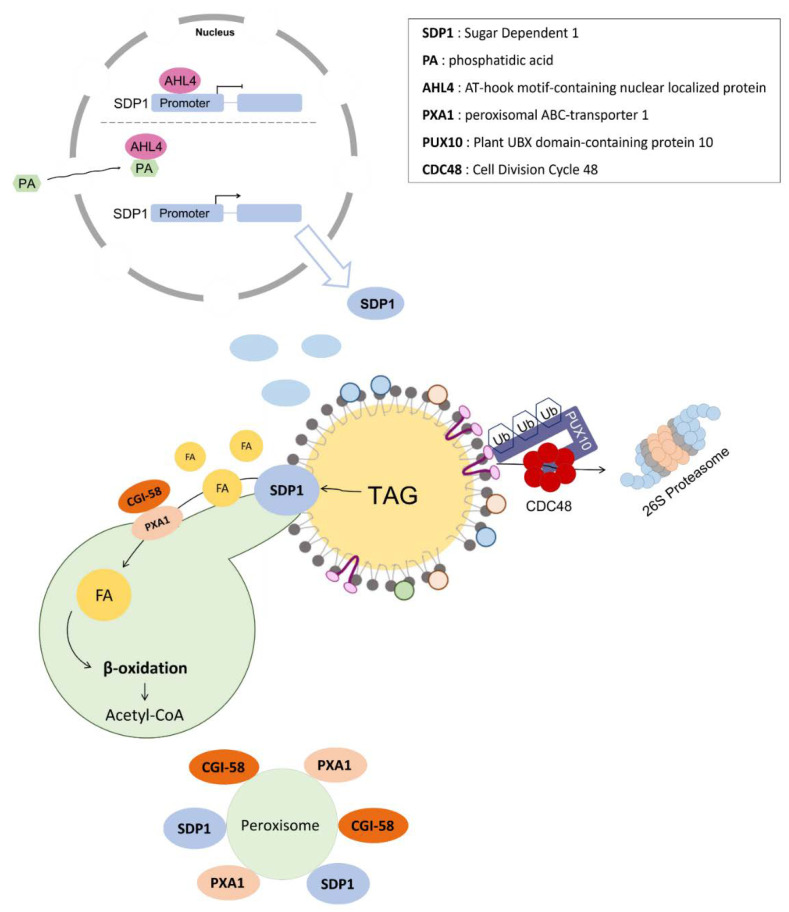
Mobilization of lipid droplets by plant lipases. AHL4, AT-hook motif-containing nuclear localized protein 4; SDP1, sugar-dependent 1; PA, phosphatidic acid; CGI-58, comparative gene identification-58; PXA1, peroxisomal membrane ATP-binding cassette (ABC) transporter 1; PUX10, plant UBX-domain containing protein 10; CDC48, cell division cycle 48.

Rapid turnover of TAG stored in LDs occurs during seed germination, which is essential to produce the energy required for seedling establishment. This process is facilitated by the action of LD-associated lipases that hydrolyze TAGs to release glycerol and FAs. The FAs are then catabolized by β-oxidation to generate acetyl-CoA in the peroxisomes [65]. Before TAGs in LDs are mobilized in the seeds, the degradation of the structural protein oleosin is necessary (Figure 2). An integral LD protein, plant UBX-domain containing protein 10 (PUX10), interacts with ubiquitin and the AAA ATPase cell division cycle 48A (CDC48A), which mediates the dislocation and degradation of oleosins through the ubiquitin-proteasome pathway [66]. Additionally, in *pux10* knockout mutants, the size of LDs significantly increases because of the delayed degradation of LD proteins, such as oleosins [67].

Sugar-dependent 1 (SDP1), a patatin-like protein, is the primary lipase involved in TAG catabolism during seed germination (Figure 2) [68,69]. *Arabidopsis sdp1* mutant seeds exhibit impaired TAG breakdown, leading to delayed post-germination growth [68]. SDP1 also participates in TAG hydrolysis in the leaves and roots of adult plants [70,71]. The inactive form of SDP1 is localized to the cytoplasmic surface of the peroxisomal membrane during the early stages of post-germination growth, which then moves to the LD surface to mediate TAG hydrolysis via peroxisomal extensions termed peroxules. (Figure 2) [72,73]. The association between LDs and peroxisomes is characterized by the enlargement of peroxisomes during storage oil mobilization [74] and is mediated by PXA1, a peroxisomal membrane ATP-binding cassette (ABC) transporter, which facilitates the uptake of FAs into peroxisomes [75,76]. The core retromer, which plays a role in protein trafficking, participates in the timely transfer of SDP1 from peroxisomes to LDs via peroxules [72]. The expression of the *SDP1* gene is regulated by an AT-hook motif-containing nuclear localized (AHL) protein, AHL4 (Figure 2) [77]. AHL4 binds to the promoter region of the *SDP1* gene and suppresses its transcription (Figure 2). Conversely, a lipid mediator, PA, inhibits the interaction between AHL4 and the *SDP1* promoter, thereby promoting TAG degradation (Figure 2) [77]. Therefore, an increase in the oil content of rapeseed (*Brassica napus* L.) and *Jatropha curcas* could be achieved by RNAi knockdown of *SDP1* [78,79]. In addition to SDP1, other patatin-like lipases are present in *Arabidopsis*, including SDP1-like (SDP1L) and adipose triglyceride lipase-like (ATGLL) [69]. In mammalian cells, ATGL is an essential enzyme for the release of FAs, as it catalyzes the first step in TAG lipolysis [80], and lower levels of ATGL lead to neutral lipid-storage disease [81]. However, ATGLL, the *Arabidopsis* homolog of ATGL, does not significantly contribute to TAG breakdown following seed germination; therefore, its physiological function in plants remains to be elucidated [69]. SDP1 and SDP1L have 74% sequence similarity, and SDP1L could partially complement the *sdp1* mutant. Moreover, in the *sdp1* mutant, SDP1L plays the primary role in residual TAG hydrolysis [69].

Although comparative gene identification-58 (CGI-58) is not a lipase, it plays a role as the co-activator of ATGL in mammalian cells [82]. Recessive mutations in the CGI-58 gene result in a genetic disease called Chanarin–Dorfman syndrome, which is correlated with a decrease in lipolytic events in multiple tissues [83]. Although little is known about lipases that are affected by CGI-58 in plants, the disruption of the *Arabidopsis* homolog of *CGI-58* caused a dramatic increase in TAG levels and resulted in the accumulation of Chanarin–Dorfman-like LDs [76,84]. In *Arabidopsis*, CGI-58 is localized in the cytosol and on the surface of peroxisomal membranes, where it interacts with, and positively regulates, PXA1 activity in non-seed tissues, which consequently stimulates the transport of FAs into peroxisomes for their catabolism by β-oxidation [76].

Recently, oil body lipase 1 (OBL1), a lipase that hydrolyzes TAG in pollen tubes, has been identified in *Arabidopsis* [85]. *AtOBL1* is expressed in pollen tubes and seedlings; however, when overexpressed in the leaves of *Nicotiana benthamiana*, both AtOBL1 and NtOBL1 (*N. benthamiana* OBL1) were localized to LDs. AtOBL1 displayed lipase activity with TAG, diacylglycerol, and 1-monoacylglycerol. Furthermore, the *obl1* mutant exhibited impaired pollen tube growth, indicating that TAG degradation in pollen tubes drives their growth [85]. In addition, *AtSDP1L* is also highly expressed in pollen grains, suggesting that AtSDP1L also plays a role in mobilization of LDs for pollen germination [69].

## 4. Conclusions and Future Perspectives

In this review, we summarized the mechanisms by which plant LDs are synthesized and TAGs in LDs are mobilized by lipases. The LDs play critical roles in various aspects of plant physiology, other than energy storage. Therefore, in the future, it is essential to investigate how the metabolism of LDs contributes to the multiple stages of plant development, responses to biotic and abiotic stresses, and establishment of plant biomass in both seed and non-seed tissues. In addition, uncovering the regulatory mechanisms underlying the biogenesis and degradation of plant LDs will be advantageous, and the results of these studies could be applied to develop value-added crops that can produce oil.

## Data Availability

Not applicable.
